# Outlook for coeliac disease patients: towards bread wheat with hypoimmunogenic gluten by gene editing of α- and γ-gliadin gene families

**DOI:** 10.1186/s12870-019-1889-5

**Published:** 2019-08-01

**Authors:** Aurélie Jouanin, Jan G. Schaart, Lesley A. Boyd, James Cockram, Fiona J. Leigh, Ruth Bates, Emma J. Wallington, Richard G. F. Visser, Marinus J. M. Smulders

**Affiliations:** 1 Wageningen University and Research, Plant Breeding, Wageningen, The Netherlands; 2 The John Bingham Laboratory, NIAB, Huntingdon Road, Cambridge, UK

**Keywords:** Gene editing, CRISPR/Cas9, Mutation breeding, γ-Irradiation, Wheat, Polyploid, Gluten, α-Gliadin, γ-Gliadin, Coeliac disease

## Abstract

**Background:**

Wheat grains contain gluten proteins, which harbour immunogenic epitopes that trigger Coeliac disease in 1–2% of the human population. Wheat varieties or accessions containing only safe gluten have not been identified and conventional breeding alone struggles to achieve such a goal, as the epitopes occur in gluten proteins encoded by five multigene families, these genes are partly located in tandem arrays, and bread wheat is allohexaploid. Gluten immunogenicity can be reduced by modification or deletion of epitopes. Mutagenesis technologies, including CRISPR/Cas9, provide a route to obtain bread wheat containing gluten proteins with fewer immunogenic epitopes.

**Results:**

In this study, we analysed the genetic diversity of over 600 α- and γ-gliadin gene sequences to design six sgRNA sequences on relatively conserved domains that we identified near coeliac disease epitopes. They were combined in four CRISPR/Cas9 constructs to target the α- or γ-gliadins, or both simultaneously, in the hexaploid bread wheat cultivar Fielder. We compared the results with those obtained with random mutagenesis in cultivar Paragon by γ-irradiation. For this, Acid-PAGE was used to identify T1 grains with altered gliadin protein profiles compared to the wild-type endosperm. We first optimised the interpretation of Acid-PAGE gels using Chinese Spring deletion lines. We then analysed the changes generated in 360 Paragon γ-irradiated lines and in 117 Fielder CRISPR/Cas9 lines. Similar gliadin profile alterations, with missing protein bands, could be observed in grains produced by both methods.

**Conclusions:**

The results demonstrate the feasibility and efficacy of using CRISPR/Cas9 to simultaneously edit multiple genes in the large α- and γ-gliadin gene families in polyploid bread wheat. Additional methods, generating genomics and proteomics data, will be necessary to determine the exact nature of the mutations generated with both methods.

**Electronic supplementary material:**

The online version of this article (10.1186/s12870-019-1889-5) contains supplementary material, which is available to authorized users.

## Background

Bread wheat (*Triticum aestivum* L.) is a staple crop worldwide and an important source of calories, nutrients, fibre and protein. The largest protein fraction in wheat grains is gluten, a polymer of glutenins and gliadins. Glutenins provide elastic properties, which are essential for bread dough quality, while gliadins provide viscosity with less impact on dough quality [1].

Wheat can cause allergies and/or intolerances after consumption in susceptible individuals [2, 3]. The most common disorder is an autoimmune reaction triggered by gluten immunogenic epitopes, known as coeliac disease (CD), which occurs in 1–2% of the human population [4–8]. Currently, the only treatment is a gluten-free (GF) diet, excluding all wheat, barley and rye. On a daily basis, the GF diet is difficult to follow as wheat gluten is added to a large range of food products. In addition, GF products are less healthy, with low levels of proteins and nutrients plus high levels of salt while many additives are needed to simulate the unique rheological properties of wheat gluten [9–12]. Breeding for wheat varieties that are free of, or have reduced levels of immunogenic epitopes, is therefore a potential solution for healthier products which are safe to consume by CD patients [13–16].

Bread wheat is hexaploid, so each locus is present on the homoeologous chromosome pairs of the three different sub-genomes, A, B and D. Immunogenic epitopes occur in α- γ- and ω- gliadins and to a lesser extent in low molecular weight (LMW) glutenins while high molecular weight (HMW) glutenins are mostly safe for CD patients [17] and constitute the main gluten family responsible for bread dough quality [1]. Gliadin proteins are encoded by large gene families. They are clustered together as repetitive sequences in characterised loci, do not contain introns but do include high numbers of pseudogenes (90% in case of α-gliadins) [18]. In hexaploid wheat, the α-gliadin genes are grouped in the *Gli-2* locus on the short arm of each of the group 6 chromosomes and number between 60 and 150 copies [19, 20]. The short arm of each of the group 1 chromosomes contains both the *Gli-1* locus with around 40 γ-gliadins, and the *Gli-3* locus, with approximately 16 ω-gliadins [21], in the hexaploid genome. Variants of the immunogenic epitopes that are not recognised by T cell receptors, do exist in hexaploid bread wheat [22], but in all genotypes they are found in combination with other, highly immunogenic epitope variants [18, 23–25]. There is a correlation between the level of immunogenicity of gliadin epitopes and the sub-genome on which they are located [18, 26]; gliadins from genome B tend to be less immunogenic while gliadins from genome D are more immunogenic. Given this level of complexity, it is not surprising that conventional breeding alone has not yet succeeded in generating “gluten-safe” wheat, containing only gliadins with non-immunogenic epitopes or even with no gliadin at all [13, 16, 27].

Alternatively, RNAi targeting all three gliadin families has successfully decreased 97% of gliadin expression in bread wheat grain and the gluten extract did not stimulate CD patient T cells while dough quality was barely affected [28, 29] Similarly, Becker et al. [30] decreased the expression of 20 α-gliadins yet increased expression of other storage proteins. Wen et al. [31] reduced the expression of DEMETER, preventing DNA-methylation changes and thus repressing glutenin and gliadin gene expression in the endosperm. As the transgenic RNAi construct remains in the wheat genome to silence the genes, such plants are subject to GM regulation. Another approach towards wheat with hypoimmunogenic gluten is mutation breeding. This method which is exempted from GM regulation, has recently been applied to develop “ultra-low gluten” barley [32], which is being used to produce gluten-free beer in Germany. Developing wheat with hypoimmunogenic gluten using a similar approach is theoretically possible although it constitutes a greater challenge [15]. Wheat γ-irradiated lines have to be identified that lack large regions on the short arm of chromosomes 1 or 6, yet have lost gliadin or LMW-glutenin genes in one of the three homoeologous genomes. Plants with the 12 different events have to be self-fertilised to become homozygous for the deletion and then inter-crossed to obtain hypoimmunogenic gluten while maintaining bread wheat unique baking quality conferred by HMW-glutenin loci. Gene editing using CRISPR/Cas9 represents an alternative approach that enables modification or deletion of the immunogenic gliadin genes in order to generate non-immunogenic gluten while retaining the viscosity provided by gliadins required for good bread dough quality. CRISPR/Cas9 has successfully been used in polyploid wheat to induce mutations in all six alleles of single copy genes. Zhang et al. [33] targeted up to four genes in bread wheat, using various gene editing approaches. Gil-Humanes et al. [34] targeted two single-copy genes simultaneously for gene targeting (i.e. with a DNA repair template) without integrating a Cas9 construct in the genome. CRISPR/Cas9 has been used once before to modify gluten, when Sánchez-León et al. [16] targeted multiple α-gliadin genes.

In this study, we have transformed the hexaploid bread wheat cultivar Fielder with constructs containing Cas9 and combinations of single guide RNAs (sgRNA) targeting specific sites upstream of, or within, the CD epitopes in the α-gliadin as well as the γ-gliadin gene family. These constructs would potentially create nucleotide changes and small indels in the epitope regions, or deletion of the epitope regions, either in, or out of frame. In the ideal scenario, small in-frame mutations would be generated, producing non-immunogenic gliadins which retained the desired rheological properties. Small out of frame mutations upstream the epitope region, would generate a knock out of the gliadin that should prevent the immunogenicity but may affect bread dough quality. However, the transcription of the truncated protein could prevent the often-reported compensation by other gliadin genes [35, 36]. Since gliadin genes are clustered as repeat sequences, simultaneously targeting non-consecutive genes could generate the deletion of intervening gliadin gene copies. This possibility, similar to but more precise than γ-irradiation events, presents the advantage of fully supressing the epitopes, but often triggers compensation of expression by the remaining gliadin genes. This attempt to use CRISPR/Cas9 to mutate gliadin families in polyploid wheat was a pilot experiment. The aim was to mutate as many gliadin genes as possible, in any manner, to test whether CRISPR/Cas9 could be an appropriate method to simultaneously modify sufficient gene copies in order to change qualitatively and/or quantitatively the gliadin content in wheat grains. An additional aim was to study the frequency of the different mutation types and to identify the most relevant methods to use in order to screen for wheat grain with mutated gliadins.

Due to the high sequence complexity, variability and number of targeted gliadin genes, a simple PCR on wild type plants would give numerous bands. A CAPS assay to reveal mutations by identifying restriction site loss, would not be possible since not all sequences would carry the site. In addition, regular gene cloning and sequencing would not be an efficient pre-screening method, given the complexity of the genes, gene families and genomic loci under study. Indeed, the cloning of the 50 to 100 single different gene copies present in a plant cannot be guaranteed and would require deep sequencing for every single Fielder-CRISPR plant generated. In addition, as many gliadin genes are pseudogenes [18, 26], the actual influence of potential mutations on the phenotype could not quickly be assessed. Moreover, using this approach on cDNA would have been inappropriate at this stage since mRNA extraction would have to be done on endosperm from immature grain, without damaging the embryo required to grow the potential mutant progeny. Advanced proteomics techniques for gliadin identification are currently under development, however, being both time consuming and expensive, they would be more appropriately used as a final epitope characterisation method, rather than for pre-screening. Consequently, Acid-PAGE was employed as a first screening method to identify gliadin protein profile changes simultaneously in targeted and untargeted gliadin gene families. This method has the advantage of being cheap and revealing mutations in expressed genes with an impact on the proteome, necessary to develop wheat lines with hypoimmunogenic gluten. However, this method reveals mainly full gene copy deletions, non-sense or off-frame mutations implying absence or truncation of gene translation but not mis-sense or small in frame mutations.

We compared the changes that occurred in our Fielder-CRISPR gene-edited lines with those we found in selected lines from a γ-irradiated mutant collection in variety Paragon [37], and in selected chromosome arm deletion lines in the reference wheat cultivar Chinse Spring [38, 39].

## Results

### Sequence alignment and sgRNA design

Genomic sequences of 1273 α-gliadin and 442 γ-gliadin genes from several wheat species and varieties were translated into protein sequences and manually aligned to infer position of epitope and most conserved regions, and to superimpose this information on the DNA sequence. The alignment was optimised by hand, especially around the repetitive parts of α- and γ-gliadin protein sequences as presented in Additional file 1: Figure S1 and Additional file 2: Figure S2, respectively. These Supplementary Figures are useful to quickly visualise the species and homoeologous genome to which they are associated and the sub-grouping based on the α- and γ-gliadin sequence pattern and the position or absence of the different overlapping CD canonical epitopes.

For α-gliadins, five protein subgroups exist based on sequence patterns, two associated to genome A, two to genome B, and one that presents a unique amino-acid variant specific for genome A or D. Some CD epitopes were present or absent in specific protein subgroups. As a consequence, some CD epitopes are specific for one homoeologous genome (Additional file 1: Figure S1). Pseudogenes were identified having similar sequences to intact genes but harbouring early stop codons at various places [40].

In γ-gliadins, six protein subgroups exist based on sequence patterns, two associated with genome A, one with genome B and the three others associated to genomes B and/or D (Additional file 2: Figure S2). Some CD epitopes were only present in some specific protein subgroups. However, this does not lead to a good correlation between epitopes and a sub-genome, as most epitopes occur in γ-gliadins from all three genomes (Additional file 2: Figure S2). Six groups of pseudogenes were observed that did not simply contain an early stop codon but also had divergent sequences compared to the 6 groups of full length γ-gliadins. This is consistent with the hypothesis of Goryunova et al. [41] that the various γ-gliadin groups largely predate the evolution of the genomes within the genus Triticum/Aegilops. Consequently, sgRNA were designed focusing on full length genes while considering pseudogenes as much as possible.

**Fig. 1:**
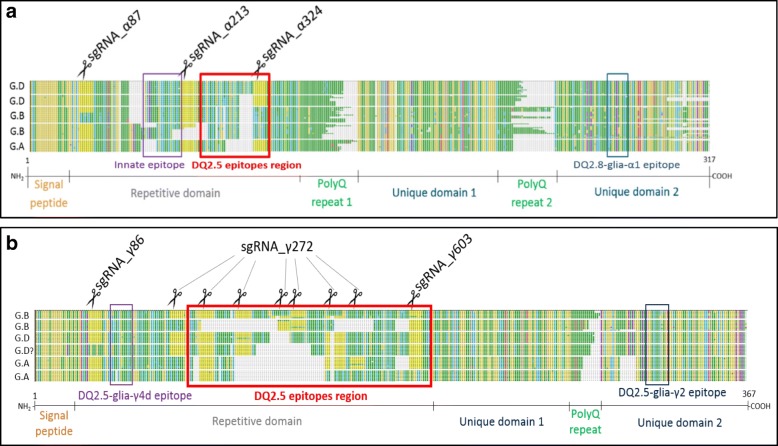
Alignment of α- and γ-gliadin protein sequences with sgRNA position and potential sites of gene editing. A representation of the protein sequence alignments of α-gliadins (**a**) and γ-gliadins (**b**) based on a total of 438 and 187 DNA sequences, respectively. The variation in the sequences form patterns which are grouped here and associated to the genome in which they are mostly found (A, B, D on the left side of each group), based on comparison of hexaploid wheat sequences with sequences from diploid relatives. The different gliadin protein domains are indicated and the position of the CD immunogenic epitopes are boxed. The DQ2.5 epitopes box includes the DQ2.5-α1, − α2 and - α3 epitopes (Additional file 1: Figure S1 and Additional file 2: Figure S2). The sgRNAs targeted motifs are highlighted in yellow and the potential gene editing sites are marked with scissors. sgRNA_γ272 may cut multiple times, depending on the number of repetitions of the most abundant γ-gliadin CD epitope, DQ2.5-glia-γ4c, which it targets. Details on the alignment, sequence patterns and CD epitopes can be found in Additional file 1: Figure S1 and Additional file 2: Figure S2. The MEGA and fasta files are also provided as Additional Files.

Six sgRNAs were designed, three of which target α-gliadins (Fig. 1a) and the other three targeting γ-gliadins (Fig. 1b). In each gene family one target site was placed upstream in the gene, soon after the signal peptide, with the aim of disrupting the open reading frame and the two others were near or in the innate or DQ2.5 coeliac disease epitope regions in order to modify or remove the epitope region.

Our alignments show that as a result of the existing sequence differences, largely within the DQ2.5 epitope region (Additional file 1: Figure S1 and Additional file 2: Figure S2), one sgRNA cannot theoretically target all the intended sites present in the hexaploid bread wheat genome. For instance, sgRNA_α324 was not predicted to target the two groups of genes associated with genome B while sgRNA_α87 was only predicted to target some genes related to one of the two α-gliadin groups of the B genome. sgRNA_α213, however, should recognise its target motif in all 5 different α-gliadins DQ2.5 groups, regardless of the genome they are associated with (Fig. 1a). For the γ-gliadins, sgRNA_γ86 may not target one of the DQ2.5 groups typical for hexaploid wheat (possibly genome D) while sgRNA_γ603 was not predicted to target a group of genes associated to genome A. sgRNA_γ272 should target most of the γ-gliadin DQ2.5 sequences and expected to cut between 1 and 6 times depending on the number of repetitions of the targeted motif, corresponding to the most abundant γ-gliadin CD epitope DQ2.5-glia-γ4c, throughout the epitope region (Fig. 1b; Additional file 2: Figure S2).

**Fig. 2:**
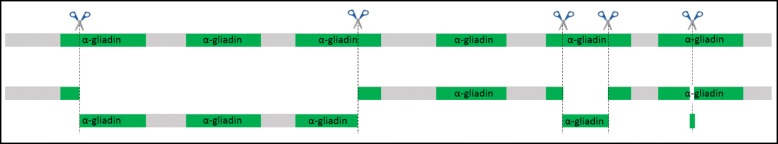
Representation of one α-gliadin *Gli-2* locus and different mutation types potentially induced by CRISPR/Cas9. This schematic α-gliadin *Gli-2* loci representation shows genes clustered and different types of mutations that can be induced by sgRNA. Simultaneous cuts in non-consecutive genes can delete the intervening genes. Similarly, two simultaneous cuts flanking the epitope can delete only this region, whilst simple small indels or base substitutions can also occur.

Multiplexing sgRNAs in clustered gene families can generate many different types of mutation (Fig. 2). Indeed, a simultaneous Cas9 cut within two non-consecutive gliadin genes can delete the intervening genes. In addition, a simultaneous Cas9 cut upstream of and downstream of the epitope region, may enable a minimal deletion of only the potentially immunogenic region. Finally, simple single cuts may create small indels or base substitutions at that site.

### Generation of CRISPR/Cas9_sgRNA constructs and transgenic fielder wheat plants

Four different CRISPR/Cas9_sgRNA binary constructs were created containing Cas9 plus different combinations of the sgRNAs (Table 1), transferred to *A. tumefaciens* and used in stable transformation experiments with immature embryos isolated from wheat cultivar Fielder. The number of transformed T0 plants regenerated and expressing the *Cas9* gene (as determined by RT-PCR on leaf samples) are presented in Table 1, together with the number of plants harbouring one or two copies of the *nptII* gene, part of the T-DNA containing the CRISPR/Cas9 construct.

**Table 1:** Summary of T0 Fielder-CRISPR regenerated plants expressing Cas9 and their nptII copy number

	T0 Fielder wheat plants		
	Regenerated	Expressing Cas9	1 or 2 transgene copies
Construct α1_sgRNA sgRNA_α87	40	38 (95%)	21 (55%)
Construct α2_sgRNA sgRNA_α213 + sgRNA_α324	32	12 (38%)	4 (33%)
Construct γ3_sgRNA sgRNA_γ86 + sgRNA_γ272 + sgRNA_γ603	36	35 (97%)	26 (74%)
Construct α2γ3_sgRNA sgRNA_α213 + sgRNA_α324 + sgRNA_γ86 + sgRNA_γ272 + sgRNA_γ603	42	32* (76%)	12 (38%)
TOTAL	150	117 (78%)	63 (54%)

For each construct, between 32 and 42 plants were regenerated. The * indicates that 34 plants expressed Cas9 but 2 were discarded since they did not contain all 5 sgRNAs. Overall, 78% of plants expressed the Cas9, among which 54% had 1 or 2 copies of the nptII selectable marker gene whereas the remainder contained additional copies of nptII.

A total of 150 T0 Fielder-CRISPR plantlets were regenerated, using the four constructs, among which 31 plants did not express the Cas9, had prematurely aborting grains, or died. Among the plants carrying the T-DNA, only two plants transformed with the α2γ3_sgRNA construct did not carry all the sgRNAs. Generally, T0 wheat plants generated 4 to 6 ears and 80–250 T1 grains. A subset of T1 grain samples was analysed using Acid-PAGE.

#### Acid-PAGE analysis of gliadin proteins

Acid-Polyacrylamide Gel Electrophoresis has been used for decades to differentiate and identify wheat varieties based on their characteristic gliadin protein profile [42]. Here, we used Acid-PAGE to identify grains with modified gliadin protein profiles from Fielder-CRISPR plants compared with wild-type Fielder, and to determine the type of changes that had occurred. We first optimised the interpretation of the gels using Chinese Spring deletion lines, and also analysed a Paragon γ-irradiated population to be able to compare the type of changes induced by irradiation mutagenesis with those induced by gene editing. Note that some C-glutenins, which have a very high sequence similarity to gliadins, may also be edited. As we used non-reducing extraction conditions, they will not be extracted and hence are not visible on the Acid-PAGE gels. This means that there may be additional edits in the plants that were screened, that are not visible on the gels.

#### Optimizing acid-PAGE analysis using Chinese spring deletion lines

Chinese Spring (CS), a model variety for hexaploid bread wheat, plus the CS nullisomic/tetrasomic and CS Kansas deletion lines, identified as either missing and/or having substitutions of gliadin genes on known homoeologous chromosomes arms, were used to set up and optimise the gliadin protein profile screening method using Acid-PAGE.

**Fig. 3:**
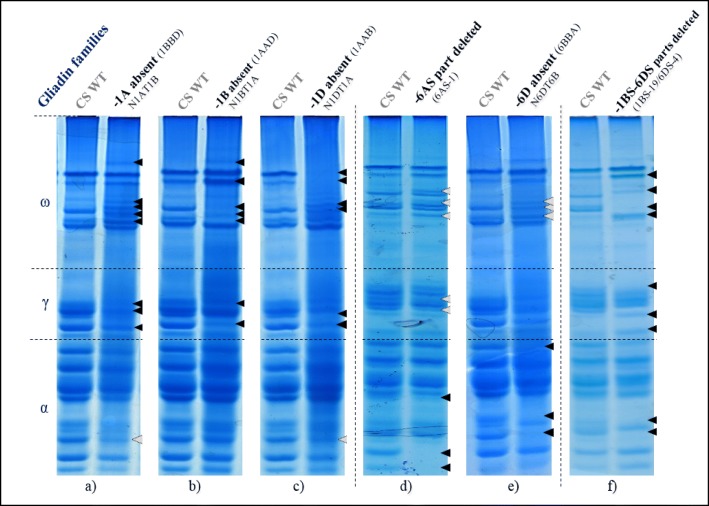
Acid-PAGE of Chinese Spring deletion lines showing altered endosperm gliadin protein profiles. Gliadin extracts from grains of Chinese Spring nullisomic/tetrasomic lines and Kansas deletion lines were run on non-denaturing Acid-PAGE alongside a gliadin extract from Chinese Spring (CS WT). The lanes displayed next to each other have been run alongside each other on the same gel but each panel represents a different gel. Each sample was always run alongside CS WT as a control. The black and grey arrows point at the changes in the protein groups from the deleted chromosome arms and in others respectively. CS gliadin profile in absence of **a** Chr1-AS, **b** Chr1-BS, **c** Chr1-DS, **d** part of Chr6-AS, **e** Chr6-DS, **f** part of Chr1-BS and Chr6-DS.

These deletion and substitution lines revealed differences in gliadin protein patterns compared with to CS. Various bands were missing or shifted on the Acid-PAGE, depending on which chromosomes (Chr1 γ- and ω-gliadins or Chr6 α-gliadins) and which homoeologous genome (A, B or D) were changed (Fig. 3). As expected, deletion lines and nullisomic/tetrasomic lines confirmed each other. For instance, in panel e) and f) of Fig. 3, the same two α-gliadin proteins are missing at the bottom of the gel. Band shifts or changes in band intensity were sometimes also seen in a gene family for which the loci had not been changed, such as ω-gliadins in case of a deletion in chromosome 6 (Fig. 3d, e). Deletion or substitution of genome 6B did not show any perceptible change, neither in the α-gliadin proteins, nor in the other gliadin families (data not shown).

In addition to optimising the method, the use of the CS deletion lines gave indications on the position of bands from α-, γ- and ω-gliadins which are specifically associated with gliadin proteins originating from A, B or D homoeologous genomes [43]. This will constitute benchmarks to infer, on non-characterised mutant lines, which homoeologous chromosome is the most likely to have been impaired.

#### Analysis of the paragon γ-irradiated population

Wheat grains from various Paragon γ-irradiated lines were screened using Acid-PAGE. Differences in gliadin proteins profiles were observed between Paragon and M4 grains in 14 out of 360 γ-irradiated lines tested (3.88%).

**Fig. 4:**
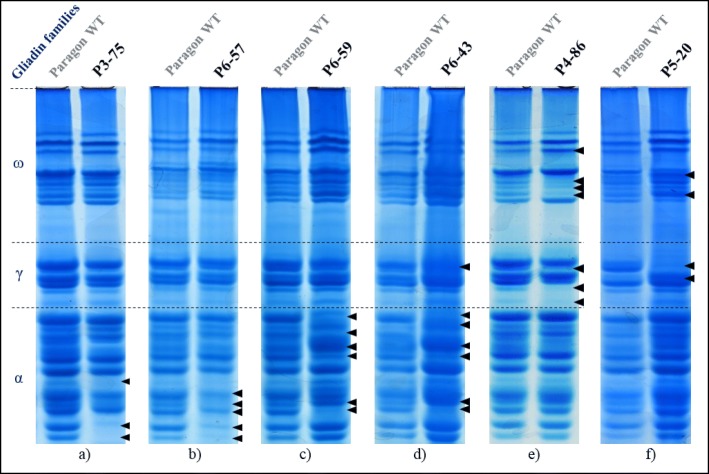
Acid-PAGE of selected Paragon γ-irradiated mutant lines that showed changes in gliadin protein profiles. Gliadin extracts from grains of the M4 generation of Paragon γ-irradiated mutant population were run on non-denaturing Acid-PAGE alongside a gliadin extract from Paragon (Paragon WT). Each panel represent a different gel. The lanes displayed next to each other have been run alongside each other. The black arrows point at the changes observed in the irradiated lines. **a** and **b** α-gliadin bands missing, probably correlated to changes in Chr6-AS, **c** and **d** α-gliadin bands changes that are different from any change observed in deletion lines and nullisomic/tetrasomic lines, **e** γ- and ω-gliadin bands missing, probably due to changes in 1BS, **f** γ-gliadin bands missing and ω-gliadin bands shifts and intensity changes, probably due to changes in Chr1-AS.

Three lines showed differences in only the α-gliadins (Fig. 4a, b, c), one line displayed variations in only the γ-gliadins while four lines presented variations in only the ω-gliadins. One line showed differences in both α- and γ-gliadins (Fig. 4d) while two lines presented changes in both γ-and ω-gliadins (Fig. 4e, f). No line screened showed variations in all three gliadin families (Table 2).

**Table 2:** γ-irradiated Paragon lines and the inferred chromosome arms underlying the gliadin protein profile variations

	Altered protein profile		
	α	γ	ω
P3-75	6AS	-	-
P4-34	-	1BS	-
P4-84	-	1BS	1BS
P4-86	-	1BS	1BS
P5-20	-	1AS	1AS
P5-53	-	-	1BS
P6-02	-	1AS	1AS
P6-43	6DS	1AS	-
P6-57	6AS	-	-
P6-59	6DS	-	-
P6-60	-	-	1AS
P6-65	-	-	1AS
P6-74	-	-	1AS
P10-73	-	1AS	1AS

Paragon γ-irradiated lines and the gliadin families in which altered protein profiles are seen on Acid-PAGE. The chromosome arms inferred to be altered by the γ-irradiation and to cause the protein profiling changes are indicated in the table. In total, four lines showed changes in the α-gliadins, seven lines had differences in the γ-gliadins and nine lines displayed variations in the ω-gliadins. P6–57 had large changes in α-gliadins from, most likely, 6AS.

By comparing the Paragon mutant gliadin protein profiles with those obtained from CS deletion lines and CS nullisomic/tetrasomic lines, it was possible to infer the homoeologous chromosome location of the gliadin genes altered by γ-irradiation mutagenesis in the identified Paragon lines (Table 2).

#### Analysis of the fielder CRISPR-Cas9 plants

**Fig. 5:**
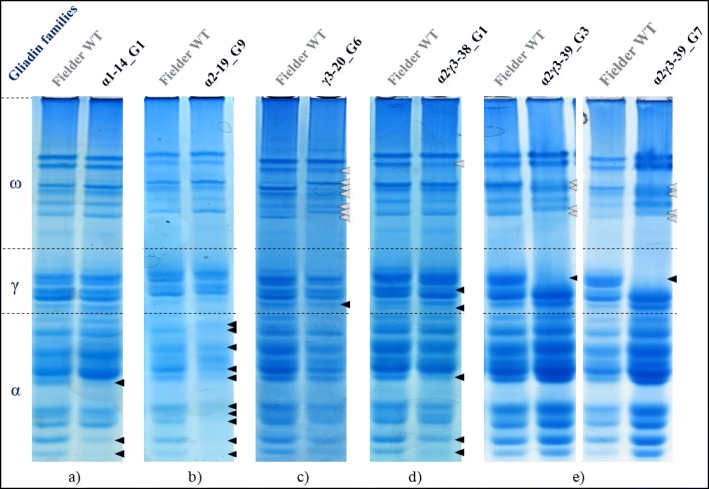
Acid-PAGE of T1 grains showing altered gliadin protein profiles. Gliadin extracts from Fielder-CRISPR T1 grain from each of the 4 constructs were run on non-denaturing Acid-PAGE alongside the gliadin extract from Fielder wild type. Each panel represent a different gel. The lanes displayed next to each other have been run alongside each other. The start of the sample names refers to the constructs with the sgRNAs they include (α1, α2, γ3 or α2γ3), followed by the T0 plant line and grain number. The black and grey arrows point respectively at the changes intended or unintended by the construct present in the plant that set the grains. **a** α-gliadin bands missing likely related to mutations on Chr6-AS, **b** α-gliadin bands missing or lower expressed likely related to mutations on Chr6 in all 3 homoeologous genomes, **c** ω-gliadin bands shifted up and γ-gliadin band with lower expression level likely related to mutations on Chr1-BS or DS, **d** ω- γ- and α-gliadin bands with lower expression likely related to mutations on Chr6-AS, e) ω-gliadin bands shifted up and γ-gliadin bands missing likely to be related to Chr1-AS, similar mutant profile in two different T1 grains from the same T0 plant.

For all of the 117 T0 plants expressing the Cas9 mRNA and carrying the full complement of expected sgRNAs, 8 or more randomly selected T1 grains per plant were screened using Acid-PAGE. Differences in the gliadin protein profile were observed between Fielder wild type and T1 grains harvested from some T0 CRISPR/Cas9 plants (Fig. 5; Table 3).

**Table 3:** Summary of T1 Fielder-CRISPR grains, and associated T0 plants, with modified gliadin protein profile

CRISPR/Cas9 constructs	T0 plants expressing Cas9	T1 grains tested on Acid-PAGE	T0 plants giving T1 mutant grains with clear changes	T1 grains with clear changes in gliadin profile	T0 plants giving T1 mutant grains with any changes	T1 grains with any changes in gliadin profile
Construct α1_sgRNA sgRNA_α87	38	325	3 (7.9%)	4 (1.2%)	3 (7.9%)	8 (2.5%)
Construct α2_sgRNA sgRNA_α213 + sgRNA_α324	12	288	7 (58.3%)	13 (4.5%)	7 (58.3%)	19 (6.6%)
Construct γ3_sgRNA sgRNA_γ86 + sgRNA_γ272 + sgRNA_γ603	35	280	10 (28.6%)	10 (3.6%)	15 (42.9%)	23 (8.2%)
Construct α2γ3_sgRNA sgRNA_α213 + sgRNA_α324 + sgRNA_γ86 + sgRNA_γ272 + sgRNA_γ603	32	256	3 (9.4%)	11 (4.3%)	11 (34.4%)	20 (7.8%)
TOTAL	117	1149	23 (19.6%)	38 (3.3%)	36 (30.8%)	70 (6.1%)

The plants and associated T1 grains tested on Acid-PAGE, as well as the number of T0 plants giving T1 mutant grains with “clear changes” or “any changes” in Acid-PAGE profiles, and the number of T1 mutant grains showing “clear changes” and “any changes”. For each construct mutations were observed. Mutations occurring in both the targeted and non-targeted gliadin families were included in “any changes” here.

Modified profiles were found for each of the 4 CRISPR/Cas9_sgRNA constructs used (Fig. 5). When a modified grain was found, most of the other T1 grains of that transformed plant showed a wild type profile. When several mutated T1 grains were found from one T0 plant, in some cases they contained similar profile modifications (Fig. 5e), in other cases the changes were different. Similarly altered gliadin protein profiles were sometimes seen in grains coming from different T0 individuals carrying the same CRISPR/Cas9_sgRNA constructs or even from different constructs (Fig. 5a, d, at α-gliadin level). Two classes of modifications were noted: “clear changes” and “potential changes”. “Clear changes” represented instances were protein bands disappeared or appeared on the gel (Fig. 5a, b, e). Bands could also be present at a different intensity (Fig. 5c, d) or have a shifted position (Fig. 5c, e) that was not always very clear and therefore recorded as “potential changes” type. The term “any changes” refer to both clear and potential changes. The changes in protein expression were mostly present in the targeted gliadin families. However, differences in intensity or shifts in size were sometimes seen in bands associated with non-targeted gliadin families (Fig. 5c, d, e). Based on the deletion of three α-gliadin bands obtained with construct α1 in grain α1–14_G1, 21 more grains from the T0 α1–14 plant were screened. However, none of them had an altered gliadin protein profile compared with Fielder.

The constructs differed significantly in mutation efficiency for any changes (Chi square, *P* = 0.015) but not for clear changes (*P* = 0.097) (Table 3). Construct α1_sgRNA had the lowest apparent mutation efficiency, with very low number of grains showing any mutations on Acid-PAGE while having the highest number of T0 plants expressing Cas9. Construct α2_sgRNA had the lowest number of T0 plants expressing Cas9 but the highest percentage of grains showing clear changes. Construct γ3_sgRNA generated the highest number of grains with any class of mutations. Construct α2γ3_sgRNA gave only 3 plants with clearly mutated grains but these plants gave three or four mutant grains each, which is the highest ratio of mutated grain per plant (Table 3).

It was observed that construct γ3_sgRNA and construct α2γ3_sgRNA produced grains which relatively often had differences in ω-gliadin expression although these constructs do not target this gliadin family. This phenomenon was rarely observed with construct α2_sgRNA and seen only once with construct α1_sgRNA.

No correlation was found between transgene copy number present in a plant and the number of grains presenting an altered gliadin protein profile, nor in the number of mutations seen per grain. Since the Cas9 expression was observed by RT-PCR, but was not quantified, no correlation can be made between its expression level and the number of gliadin protein changes observed using Acid-PAGE.

## Discussion

In this study, CRISPR/Cas9 was deployed in hexaploid bread wheat to simultaneously target multiple gliadin genes. The Cas9-induced mutations were designed to remove gliadin gene copies or disrupt their immunogenic epitopes in gliadins, so preventing them from triggering the human immune system and causing coeliac disease upon wheat gluten consumption. At the start of this study, CRISPR/Cas9 had not been used to target large clusters of repeated gene families, such as the gliadins, in polyploid crops such as hexaploid wheat. Therefore, the challenge was not only to generate such mutant plants but also to develop screening methods to identify them and characterise their mutations further.

### Gliadin alignments, sgRNA design and generation of fielder-CRISPR lines generation

In the absence of α- and γ-gliadin genomic or proteomic sequences from the hexaploid wheat cultivar Fielder used for gliadin gene editing experiments, and considering the time and resources necessary to fully clone and sequence these gene families, an alternative approach was used mainly based on publicly available data. Only seven α-gliadins from Fielder were cloned, sequenced and merged with all α-gliadin genomic sequences from GenBank/ENA. The Fielder sequences were different to one another but similar to some sequences already present in GenBank/ENA. Deduced protein sequences were manually aligned to enable inferring gliadin epitope positions and conserved regions in gliadin gene sequences across cultivars. Using this approach, it was possible to design sgRNA targeting as many gliadin genes as possible near their epitope region, not only in Fielder but also in many other different cultivars. Alignment of gliadin sequences also revealed the presence of groups based on sequences patterns, some associated to specific homoeologous genomes, similarly to what was observed by Ozuna et al. [20]. The same approach was used for γ-gliadins, although no Fielder γ-gliadins were sequenced. The association of CD epitopes and genomes of origin was possible for α-gliadins whereas it was not straightforward for γ-gliadins.

The sequences downloaded from GenBank/ENA originated from different hexaploid varieties. When sequences from one cultivar are uploaded into the database, there is no guarantee that the set of sequence variants per cultivar is complete. Furthermore, gliadin genes are present in each homoeologous genome and clustered at specific loci as multiple copy repeats, with identical or variable sequences. A gene sequence found multiple times in one cultivar, is uploaded only once as new sequence to avoid redundancy. However, if an identical sequence was found in different cultivars or in the same cultivar in different studies by different groups, it will be present several times in the database. Therefore, the frequency with which a sequence is present in the alignments generated for this study does not reflect the frequency at which a gene sequence is present in the genome of a cultivar such as Fielder. As a result, the proportion of sequences targeted by the different sgRNA designed does not reflect the real percentages of matching sequences present in Fielder, especially since the evaluation was made on 100% match while in reality, sgRNA are known to also target sequences with slightly lower similarity [44].

Four CRISPR/Cas9_sgRNA constructs, containing 1 to 5 different sgRNAs, were designed and used to target α- and/or γ-gliadin gene families, in hexaploid wheat. The constructs were stably transformed into bread wheat cultivar Fielder. A total of 117 regenerated lines expressed Cas9, regardless of which construct they carried. The T1 grains generated by these plants potentially contain mutations at a number of target sites which are segregating independently. However, it is possible that the Cas9-induced mutation is heterozygous in a cell. It is also possible that Cas9 did not cut the same genes in different cells of the T0 plant, thus generating chimerical plants or even that Cas9 remained active in the gametes after meiosis. This means that, following segregations events, each grain generated by a T0 plant could have a unique assortment of gliadin mutations. For this reason, each grain was cut into three parts, the embryo and two identical pieces of endosperm, to run further complementary types of analysis (sequencing and advanced proteomics) on the identical fractions of endosperm isolated from the same T1 or T2 grain.

### Pre-screening for wheat grains with mutated gliadins

Due to the high complexity of the large gliadin gene families that are only expressed in wheat grains, the traditional screening methods such as restriction site loss or sequencing are not appropriate for pre-screening and identification of potential mutants. Instead, these methods would be relevant for in depth study and characterisation of the mutations occurring in interesting mutants identified using different pre-screening methods. Acid-PAGE was successful for identifying gliadin protein profile differences [42] and we therefore employed it for high-throughput pre-screening of the grains from mutant wheat plants. This method was first optimised using CS and associated set of deletion lines and nullisomic-tetrasomic lines. These lines were previously characterised as missing chromosomes or chromosome arms that carry gliadin genes from specific homoeologous genome. These lines enabled identifying the homoeologous chromosome arms and the sub-genome that most likely was altered in a mutant, based on the absence or shift in position of the band.

The optimised Acid-PAGE protocol was then used to identify 14 lines out of 360 (3.9%) Paragon γ-irradiated lines that showed gliadin proteins expression changes compared to wild type Paragon. Lines showed changes in one or two gliadin families but never in all three families simultaneously. Irradiation mutation is known to trigger large deletions, up to several mega-bases, which can explain the deletion of a complete gliadin gene locus. The nature of the homoeologous chromosomes altered by γ-radiation were inferred based on the results obtained using the CS deletion line resources, since similarly large deletions are expected in both line sets. The percentage of mutations observed in Paragon γ-irradiated germplasm using on Acid-PAGE was around 4%, for all visible changes in the three large gliadin gene families, each counting more than dozens of members.

Similarly, Acid-PAGE allowed pre-screening of T1 germplasm generated via CRISPR/Cas9 gene editing. Changed gliadin profiles were observed with each construct, implying that each construct contained at least one sgRNA that successfully generated mutations in some copies of the targeted α- or γ-gliadin gene family. More importantly, it indicated that CRISPR/Cas9 can edit a sufficient number of genes within large family in polyploid plants to actually generate a different phenotype in the progeny. The Acid-PAGE analysis of the Fielder-CRISPR lines revealed differences in gliadin profiles in 70 T1 grains (6.1% of the total number of grains tested) harvested from 36 T0 plants (30.8% of the total number of T0 plants expressing CRISPR/Cas9 constructs) across all four CRISPR/Cas9_sgRNA constructs used. Therefore, a single T0 plant that produced T1 mutant grains, gave on average 2 mutant grains out of 8 grains tested.

Constructs targeting γ-gliadins sometimes triggered a shift of protein bands in the untargeted ω-gliadins. This phenomenon could be explained by the deletion of ω-gliadins located between targeted γ-gliadins since both gene families are suspected to have some overlap on the short arm of chromosome 1 [45]. Moreover, it is also known that knocking out some gene in a gluten family triggers the compensation by other gene families [35, 36].

Variation in gene editing efficiency could be observed between the constructs, with construct α1_sgRNA being significantly less efficient. This could be explained by the presence of only one sgRNA in this construct while several are present in the other constructs used. It could also be due to a presence of secondary structure in the sgRNA – identified after use, using RNAfold software - that could decrease the target binding efficiency. The number of sgRNA in a construct appears to slightly increase the ratio of mutant grains obtained, but no direct correlation was observed. Indeed, construct α2_sgRNA and construct γ3_sgRNA generated 19 and 23 mutant grains respectively while construct α2γ3_sgRNA – combining both previous sgRNA guides in a single construct - gave only 20 mutant grains, which does not show a cumulative efficiency. However, estimation of the Cas9 expression level in different plants as well as the actual number of mutations generated at the DNA level would be necessary to give a robust answer regarding the variation of efficiency of the different constructs.

### Comparison with other groups also using CRISPR/Cas9 to target gluten genes in hexaploid wheat

Sánchez-León et al. [16], who used a sgRNA, namely sgRNA_α2, having 13 overlapping bases with our sgRNA_α213 and targeting α-gliadins 6 nucleotides upstream, reported a higher success rate with T1 grains from one T0 CRISPR/Cas9 line being mutated in a similar way, and inheriting those mutations in T2 grains. The difference in results may be related to a low Cas9 efficiency in this case. Cas9 sequences have quickly been improved to increase the mutation efficiency and the Cas9 version we used (different from the one used by Sánchez-León et al. [16]) has been reported as having a low efficiency [46, 47]. In addition, we used the rice actin promotor, which has previously been shown to create stable heritable edits in wheat [47], whereas most researchers have used the maize ubiquitin promoter to express Cas9 in monocot plants [16, 46]. These factors could contribute to the low number of T1 mutant grains per T0 plants obtained but also the low level of gene copies mutated within each mutant wheat grain. It could also explain the absence of mutated phenotype inheritance, assuming that with a higher efficiency and most of the targeted genes mutated, compensation of the mutated gene copies becomes much more difficult for the crop.

### Comparison of CRISPR/Cas9 targeted mutations with random mutagenesis using γ-irradiation

Using either CRISPR/Cas9 or γ-irradiation, the mutation efficiency revealed by the Acid-PAGE is comparable. However, the actual mutation rate obtained by using CRISPR is probably much higher. Fielder-CRISPR lines tested correspond to T1 generation grain potentially heterozygous for mutations while Paragon γ-irradiated lines represented M4 generation grain where most mutations are homozygous and easier to visualise. In addition, Acid-PAGE will probably not reveal amino-acid substitutions or small in-frame indels potentially generated by CRISPR/Cas9 since proteins with different sequences but having similar molecular weight/charge ratio can be represented by a single band on the gel [48]. Moreover, knocking out a gliadin gene may not supress any protein band if another similar gene has not been knocked out. Alternatively, 2D-gels could give a higher resolution but other methods such as deep DNA sequencing are needed to reveal and characterise more subtle types of mutations and to get a better idea of the differences in efficiency between both methods.

Interestingly, some T1 CRISPR-Fielder grains showed an altered gliadin protein profile similar to some M4 Paragon γ-irradiated grains (Fig. 5a, e and Fig. 4a, f). The difference, however, is that in the Fielder-CRISPR grains, only the α- or γ-gliadin gene family were targeted and are probably modified whereas in the Paragon γ-irradiated grains it is likely that gliadin genes as well as unrelated flanking genes were deleted. Furthermore, wheat lines with small deletions in the epitope region possibly generated by CRISPR/Cas9 would be more favourable than multiple gene copy deletions generally generated by γ-irradiation for two reasons. First of all, the gliadin copies would remain, but in a ‘safer’ form for CD, while retaining the baking properties that would likely otherwise be lost if the genes were totally removed using irradiation. In addition, having small CRISPR-mediated deletions might avoid gene expression compensation by other potentially immunogenic genes, whose expression is triggered when expression of a gliadin gene family is partly knocked down. Moreover, the γ-irradiated lines may have multiple unwanted deletions at other genomic loci. However, following current regulation regarding gene-editing and mutation breeding, the Fielder-CRISPR wheat could not be grown in many countries yet due to region-specific strict GM regulation while the Paragon γ-irradiated wheat could directly be grown and used in breeding programs without any restrictions [14].

## Conclusion

This pilot study aimed to mutate the large α- and γ-gliadin gene families in hexaploid wheat to decrease gluten immunogenicity for coeliac patients. We succeeded in generating wheat grains that contained gliadin protein profiles altered for the targeted gene family in the bread wheat cultivar Fielder by targeted mutagenesis using CRISPR/Cas9 and in identifying them in Paragon germplasm induced by random mutagenesis using γ-irradiation. As many gliadin genes appear likely not to have been altered, optimisation via use of different promoter and different Cas9 genes or new Base Editor systems is required to obtain wheat plants which are safe for CD patients.

The pre-screening and identification of the mutations was performed using Acid-PAGE, which identifies mostly the non-sense mutations and large deletions. However, the final purpose is to modify the epitopes into safe versions without knocking out the complete gliadin genes, in order to avoid compensation by other gliadins and to maintain baking quality. Therefore, reliable high throughput methods will be important for small in-frame mutation detection in the known CD epitopes [49]. Methods such as droplet digital PCR, enrichment and sequencing as well as advanced proteomics studies will be needed to identify also these subtle modifications and characterise them further. This should further increase the percentage of plants in which mutations have been induced, even further and enable the identification of mutations in T2 plants. Enrichment and sequencing of our gene-edited grains is described in [50].

Segregating out the CRISPR/Cas9 construct from promising lines and subsequently self-pollinating these wheat lines to make all mutations homozygous would be the next steps. Potentially interesting lines would then need extensive investigation including immunological tests using monoclonal antibodies to determine their immunogenicity level and rheological studies to evaluate the bread dough quality obtained using these generated “hypoimmunogenic-gluten” wheat lines.

## Methods

### Gliadin sequence alignment

Over 438 α-gliadin gene sequences from 30 wheat accessions and 187 γ-gliadin gene sequences from hexaploid wheat were downloaded from GenBank/ENA in September 2014 and July 2015 respectively, translated into amino acid sequences, manually aligned using Mega_6 and clustered based on their combination of known CD epitopes [49]. To deduce the sub-genome of origin of different epitopes, which is linked with the level of immunogenicity, sequences from diploid bread wheat ancestors *Triticum monococcum* and *Triticum urartu* (A genome), *Aegilops speltoides* (for the B genome), *Aegilops tauschii* (D genome) and tetraploid durum wheat *Triticum turgidum* (genome AB) were added that were present in GenBank/ENA. To complete the alignments and facilitate sgRNA design, primers F: 5′-ATGAARACMTTTCYCATC-3′ [25] and R: 5′-YAGTTRGTACCRAAGATGM-3′ were used to clone and sequence seven intact α–gliadin genes from Fielder, the spring wheat variety used for transformation. These α-gliadin gene sequences were similar to the one already present in the databases. This increased the total number of sequences used to 1273 α-gliadins and 442 γ-gliadins.

### sgRNA protospacer design

Six sgRNA protospacers were designed on conserved regions that were identified based on the sequence alignments and were present in the sequences of the hexaploid wheat cultivar Fielder. Of these six sgRNAs, three targeted α-gliadins and the other three targeted γ-gliadins (Fig. 2). In each case, one targeted a region downstream of the signal peptide, the other two targeted regions in or near epitopes. These sgRNA target the complementary DNA strand and should therefore be reverse-complemented to be found in the GenBank/ENA sequences.

sgRNA_α87: 5′-GATTTTGTGGCTGCAATTG-3′ targets α-gliadins downstream the signal peptide, P87.

sgRNA_α213: 5′-ATGGTTGTTGTGATGGAAA-3′ targets α-gliadins upstream the epitope region, P213.

sgRNA_α324: 5′-GTTGTGGTCGAAATGGTTG-3′ targets α-gliadins downstream the epitope region, P324.

sgRNA_γ86: 5′-TTGTTGTGGCCATTGTACT-3′ targets γ-gliadins downstream the signal peptide, P86.

sgRNA_γ272: 5′-AATGGTTGTTGTGGTTGCTG-3′ targets γ-gliadins within the epitope region, P274.

sgRNA_γ603: 5′-TGCTGGGGGAATGATTGTTG-3’ targets γ-gliadins downstream the epitope region, P603.

These sgRNA protospacers where tested in silico for the absence of off-targets using BLAST in the Ensembl! plant wheat database, for absence of secondary structures using RNAfold web server and for absence of cross-dimers between multiplexed sgRNAs using ThermoFisher Scientific Primer Analyzer.

Each sgRNA, including a wheat-optimised U6 promoter, gliadin-specific protospacer and sgRNA scaffold sequence, flanked by multiple unique restriction sites, was individually synthesised by GenScript.

### CRISPR/Cas9-sgRNA constructs

Type II-A *Streptococcus pyogenes* 2NLS-Cas9 gene, codon-optimized for rice and wheat expression, was cloned from the pJIT163-2NLS-Cas9 plasmid [51]. Its ribosome binding site (RBS) “CACC” was mutated into “CCACC”, using site directed mutation PCR approach, for increased expression in wheat. The optimised 2NLS-Cas9 gene plus CaMV terminator sequence was transferred into binary vector pSC4Act-R1R2-SCV [52], containing a rice actin promoter to drive the expression of the Cas9 gene. Each sgRNA, was combined using multiple unique restriction sites and then integrated into the final binary plasmids (Fig. 1) (named pAJ2_ followed by sgRNA names).

Four T-DNA constructs were produced by combining different sgRNAs, using multiple unique restriction sites:

Construct 1α_sgRNA = sgRNA_α87.

Construct 2α_sgRNA = sgRNA_α213 + sgRNA_α324.

Construct 3γ_sgRNA = sgRNA_γ86 + sgRNA_γ272 + sgRNA_γ603.

**Fig. 6:**
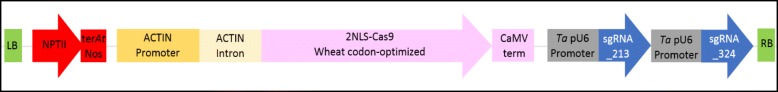
CRISPR/Cas9 T-DNA construct 2α _sgRNA. Construct 2α_sgRNA that contains sgRNA_ α213 and sgRNA_ α324, as an example of the four T-DNA constructs generated. They are similar, only the number and nature of sgRNA integrated are different.

Construct 2α3γ_sgRNA = sgRNA_α213 + sgRNA_α324 + sgRNA_γ86 + sgRNA_γ272 + sgRNA_γ603 (i.e., it combines Construct 2α_sgRNA and Construct 3γ_sgRNA). Construct 2α_sgRNA is shown as an example in Fig. 6.

### Stable transformation and generation of fielder-CRISPR plants and derived grains

The CRISPR/Cas9-sgRNA binary vectors were transferred to *A. tumefaciens* and used to transform immature embryos of Fielder [53, 54]. Selection of transformed tissues was based on the presence of the *nptII* gene conferring resistance to the G418 antibiotic. Regenerated T0 plantlets were transferred to soil and tested for T-DNA copy number using an *nptII*-based qPCR assay [54], for presence of Cas9 gene and all sgRNA using PCR, and whether Cas9 was expressed using RT-PCR. Plantlets positive in all these tests were grown in climate-controlled growth chambers, bagged during anthesis, and T1 grains harvested.

The individual T1 grains were first cut transversally, alongside the embryo, so that this could be germinated later. The resulting endosperm section was then cut in half longitudinally. One half was used for Acid-PAGE. Selected T1 embryos were germinated in Petri dishes containing filter paper soaked with water. Seedlings were potted in compost after sufficient root development and transferred to growth chambers until grain set. Ears containing T2 grains were harvested individually.

### Other plant materials

Chinese Spring (CS) wild type (WT) and selected CS Kansas deletion lines [39, 55] that lacked parts of chromosome 1 or 6, were obtained from Kansas State University (Table 1). In addition, CS nullisomic/tetrasomic lines [38] were obtained from the SeedStor, John Innes Centre, UK (Table 1). The CS lines were used to set up and optimise the Acid-PAGE separation.

The Paragon γ-irradiated population [37], obtained from JIC (Norwich, UK) based on hexaploid spring wheat cultivar Paragon, was also screened. Mature grains from a subset of 360 lines self-pollinated for 4 generations (M4) were analysed on Acid-PAGE. The lines screened were numbered from P3–47 to P6–79 and from P10–19 to P10–96 (Table 4).

**Table 4:** List of *Triticum aestivum* lines used to set up the screening method and subsequent comparison

*Triticum aestivum*	Lines	Description
Cultivar 'Chinese Spring' (CS)	WT	Control
	1AS-1	Kansas Deletion lines missing part of Chr 1 short arm that contain *Gli-1* and/or *Gli-3* loci where γ- and/or ω-gliadin genes are located
	1AS-3	
	1BS-9	
	1BS-10	
	1DS-1	
	1DS-5	
	6AS-1	Kansas Deletion lines missing part of Chr 6 short arm that contain *Gli-2* loci where α-gliadin genes are located
	6BS-4/5BS-2	
	6DS-4	
	6DS-4/1BS-19	
	N1AT1D = 1DDB	Nullisomic-tetrasomic lines with one homoeologous pair of Chr 1 that contain *Gli-1* and/or *Gli-3* loci where γ- and/or ω-gliadin genes are located substituted by another homoeologous pair of Chr 1
	N1AT1B = 1BBD	
	N1DT1A = 1AAB	
	N1DT1B = 1BBA	
	N1BT1D = 1DDA	
	N1BT1A = 1AAD	
	N6AT6D = 6DDB	Nullisomic-tetrasomic lines with one homoeologous pair of Chr 6 that contain *Gli-2* loci where α-gliadin genes are located substituted by another homoeologous pair of Chr 6
	N6AT6B = 6BBD	
	N6DT6A = 6AAB	
	N6DT6B = 6BBA	
	N6BT6D = 6DDA	
Cultivar 'Paragon'	WT	Control
	Line P3-47 to P6-79 and	360 γ-irradiated lines M4 generation
	Line P10-19 to P10-96	

### Acid-polyacrylamide gel electrophoresis (acid-PAGE)

For each Fielder-CRISPR plant, a minimum of 8 T1 grains were screened individually, alongside Fielder WT. The grain samples were loaded in duplicate. As Fielder exhibit some heterogeneity in its storage protein profile on Acid-PAGE, grains from multiple Fielder plants were loaded alongside on the gel. For T2, four grain samples from four different ears were loaded in duplicates. A similar procedure was applied for screening of CS and Paragon, with the exception that only two grains were tested as they are homogeneous for the mutations due to several generations of self-pollination.

Each half endosperm sample was crushed into fine powder, and the gliadin fraction extracted overnight at 4 °C, in 150 μl of 25% dichloroethanol solution containing 0.05% Pyronin Y. Duplicate 30 μl samples were loaded in wide slots on 13% polyacrylamide gels (acrylamide-bis 19:1) and run at 180 V, for 4 h at room temperature (adapted from [41]). Gels were stained overnight in 10:1 solution of 15% trichloroacetic acid (TCA): industrial methylated spirits (IMS) containing 10 g/L Coomassie Brilliant Blue G250, then destained overnight in water.

## Additional files

Additional file 1: **Figure S1.** α-gliadin protein sequence alignment (as image). A selected subset of the 1273 aligned α-gliadin protein sequences from cultivated wheat and wild relative is indicated here. Five patterns based on variation at the DQ2.5 epitope region were identified and are separated by horizontal dash-lines. Each pattern has been associated to a dominant genome, indicated by large A, B or D letters on the first column, with which its sequences appear to be associated to. Within a pattern, sequence originating from different wheat species diploid, tetraploid or hexaploid are separated by an empty line and the genomes present in the species are indicated by smaller A, B and D letters in the second column. The canonical sequences of CD epitopes, which often overlap with one another, are framed in different colours with their category number indicated as well. On top of the figure, scissors indicate the position at which the sgRNA designed are cutting, relatively to the position of the CD epitopes. Note that some patterns have specific CD epitope combinations and are clearly associated to a genome, while others are not. The 5th pattern has actually an amino-acid substitution in genome A compared to genome D, making it safer for CD patients. Sequences from genome B have naturally occurring amino-acid deletions within the epitope regions that prevent their recognition by the immune system in comparison to proteins from other genomes. The MEGA and fasta files for this alignment are provided as Additional file 3 and Additional file 5. (PNG 1050 kb)

Additional file 2: **Figure S2.** γ-gliadin protein sequence alignment (as image). A selected subset of the 1273 aligned γ-gliadin protein sequences from cultivated wheat and wild relatives is indicated here. Six main patterns based on variation at the DQ25 epitope region were identified and are separated by horizontal dash-lines. Each pattern has been associated to a dominant genome, indicated by large A, B or D letters on the first column, with which its sequences appear to be associated to. Within a pattern, sequence originating from different wheat species diploid, tetraploid or hexaploid are separated by an empty line and the genomes present in the species are indicated by smaller A, B and D letters in the second column. The canonical sequences of CD epitopes, which often overlap with one another, are framed in different colours with their category number indicated as well. On top of the figure, scissors indicate the position at which the sgRNA designed are cutting, relatively to the position of the CD epitopes. A clear distinction was made by a thick dash line between the full-length protein and truncated ones, usually arising from pseudogenes. Note that some patterns have specific CD epitope combinations and are clearly associated to a genome, while others are not. The MEGA and fasta files for this alignment are provided as Additional file 4 and Additional file 6. (PNG 827 kb)

Additional file 3: α-gliadin protein sequences alignment. (FAS 3386 kb)

Additional file 4: γ-gliadin protein sequences alignment. (FAS 1278 kb)

Additional file 5: α-gliadin protein sequences alignment. (DOCX 2533 kb)

Additional file 6: γ-gliadin protein sequences alignment. (DOCX 2663 kb)

## Data Availability

All sequence data analysed during this study are included in this published article and its Supplementary files.

## Abbreviations

Acid-PAGE: Acid-Polyacrylamide Gel Electrophoresis
CD: Coeliac disease
CRISPR: clustered regularly interspaced short palindromic repeats
GF: Gluten-free
gRNA: Guide RNA
HMW glutenins: High molecular weight glutenins
LMW glutenins: Low molecular weight glutenins
