# Supplementary figures and images for: Outlook for coeliac disease patients: towards bread wheat with hypoimmunogenic gluten by gene editing of α- and γ-gliadin gene families
Source: BMC Plant Biol. 2019 Aug 1;19:333. doi: 10.1186/s12870-019-1889-5 (PMC6670228; doi:10.1186/s12870-019-1889-5)

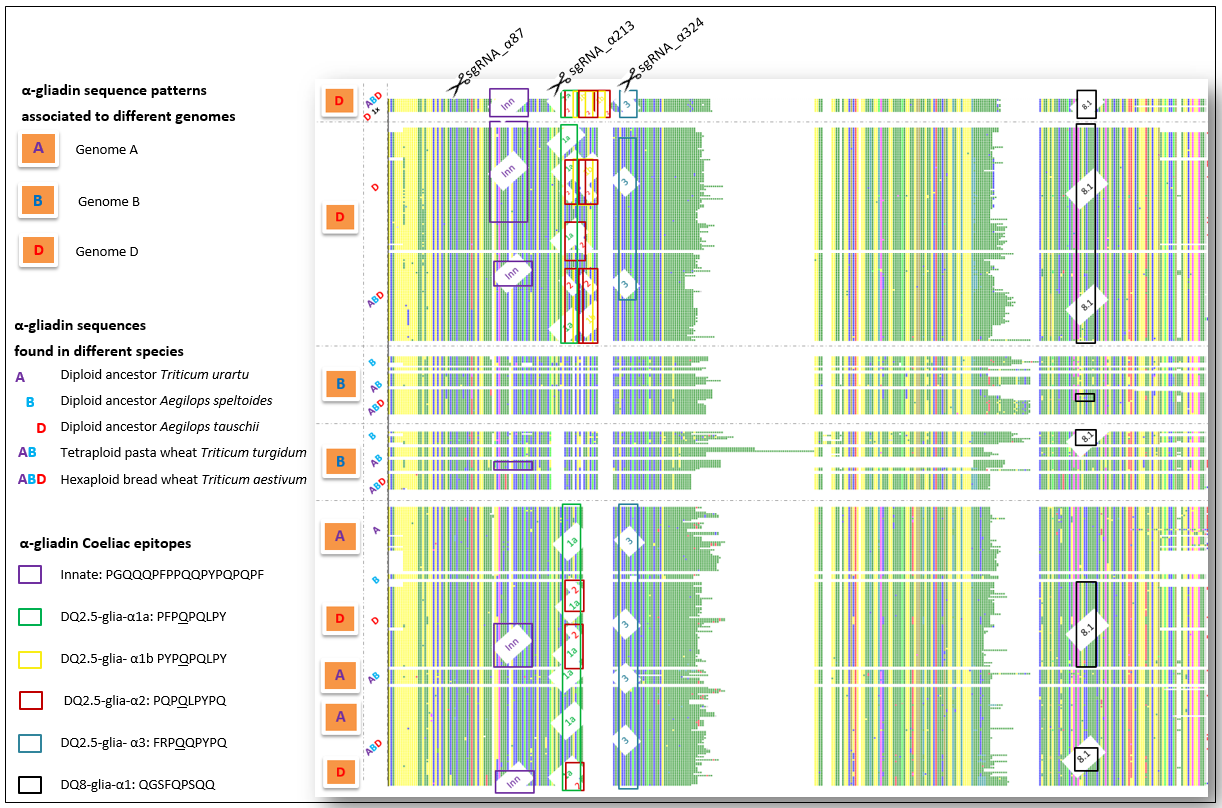

Supplement: Supplementary file 1 — Figure S1. α-gliadin protein sequence alignment (as image). A selected subset of the 1273 aligned α-gliadin protein sequences from cultivated wheat and wild relative is indicated here. Five patterns based on variation at the DQ2.5 epitope region were identified and are separated by horizontal dash-lines. Each pattern has been associated to a dominant genome, indicated by large A, B or D letters on the first column, with which its sequences appear to be associated to. Within a pattern, sequence originating from different wheat species diploid, tetraploid or hexaploid are separated by an empty line and the genomes present in the species are indicated by smaller A, B and D letters in the second column. The canonical sequences of CD epitopes, which often overlap with one another, are framed in different colours with their category number indicated as well. On top of the figure, scissors indicate the position at which the sgRNA designed are cutting, relatively to the position of the CD epitopes. Note that some patterns have specific CD epitope combinations and are clearly associated to a genome, while others are not. The 5th pattern has actually an amino-acid substitution in genome A compared to genome D, making it safer for CD patients. Sequences from genome B have naturally occurring amino-acid deletions within the epitope regions that prevent their recognition by the immune system in comparison to proteins from other genomes. The MEGA and fasta files for this alignment are provided as Additional file 3 and Additional file 5. (PNG 1050 kb) [file 12870_2019_1889_MOESM1_ESM.png]

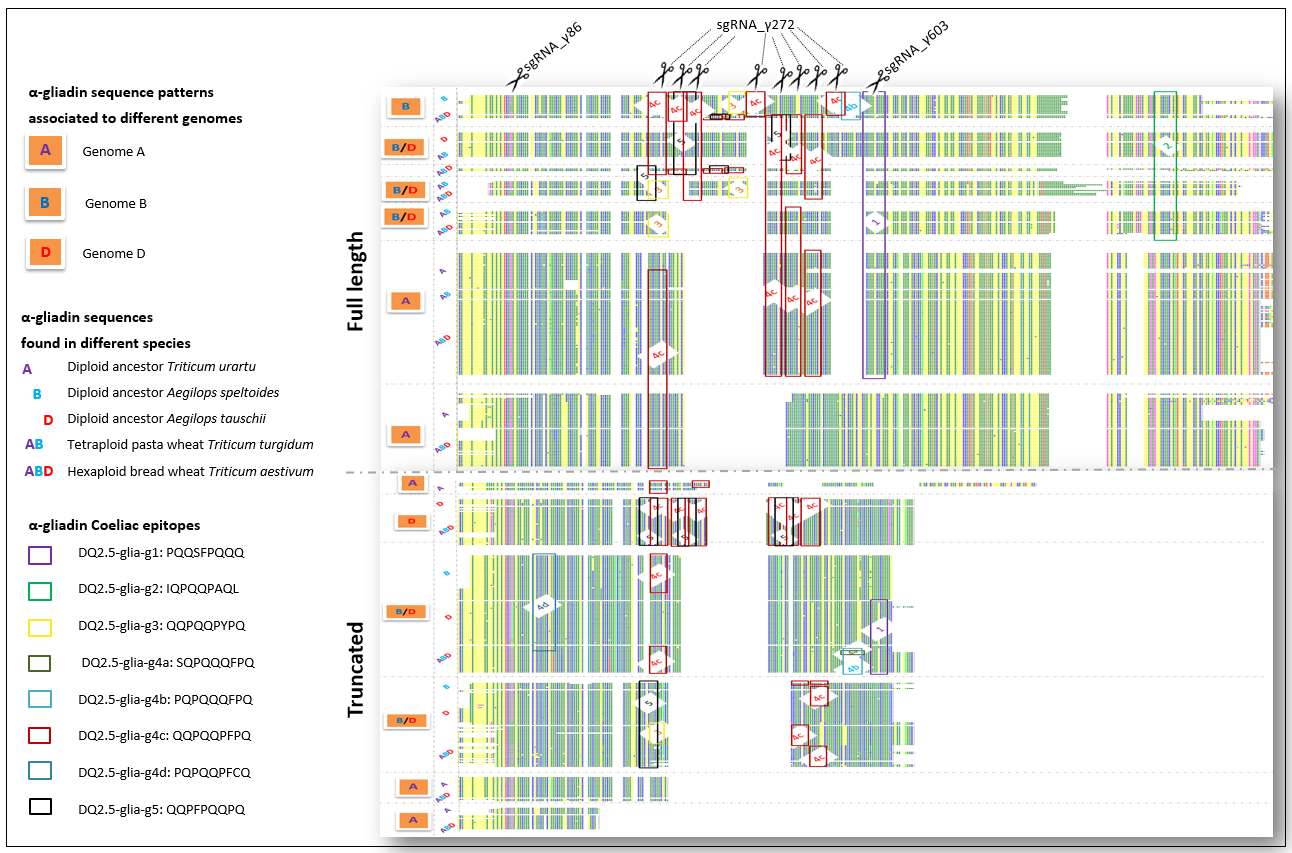

Supplement: Supplementary file 2 — Figure S2. γ-gliadin protein sequence alignment (as image). A selected subset of the 1273 aligned γ-gliadin protein sequences from cultivated wheat and wild relatives is indicated here. Six main patterns based on variation at the DQ25 epitope region were identified and are separated by horizontal dash-lines. Each pattern has been associated to a dominant genome, indicated by large A, B or D letters on the first column, with which its sequences appear to be associated to. Within a pattern, sequence originating from different wheat species diploid, tetraploid or hexaploid are separated by an empty line and the genomes present in the species are indicated by smaller A, B and D letters in the second column. The canonical sequences of CD epitopes, which often overlap with one another, are framed in different colours with their category number indicated as well. On top of the figure, scissors indicate the position at which the sgRNA designed are cutting, relatively to the position of the CD epitopes. A clear distinction was made by a thick dash line between the full-length protein and truncated ones, usually arising from pseudogenes. Note that some patterns have specific CD epitope combinations and are clearly associated to a genome, while others are not. The MEGA and fasta files for this alignment are provided as Additional file 4 and Additional file 6. (PNG 827 kb) [file 12870_2019_1889_MOESM2_ESM.png]

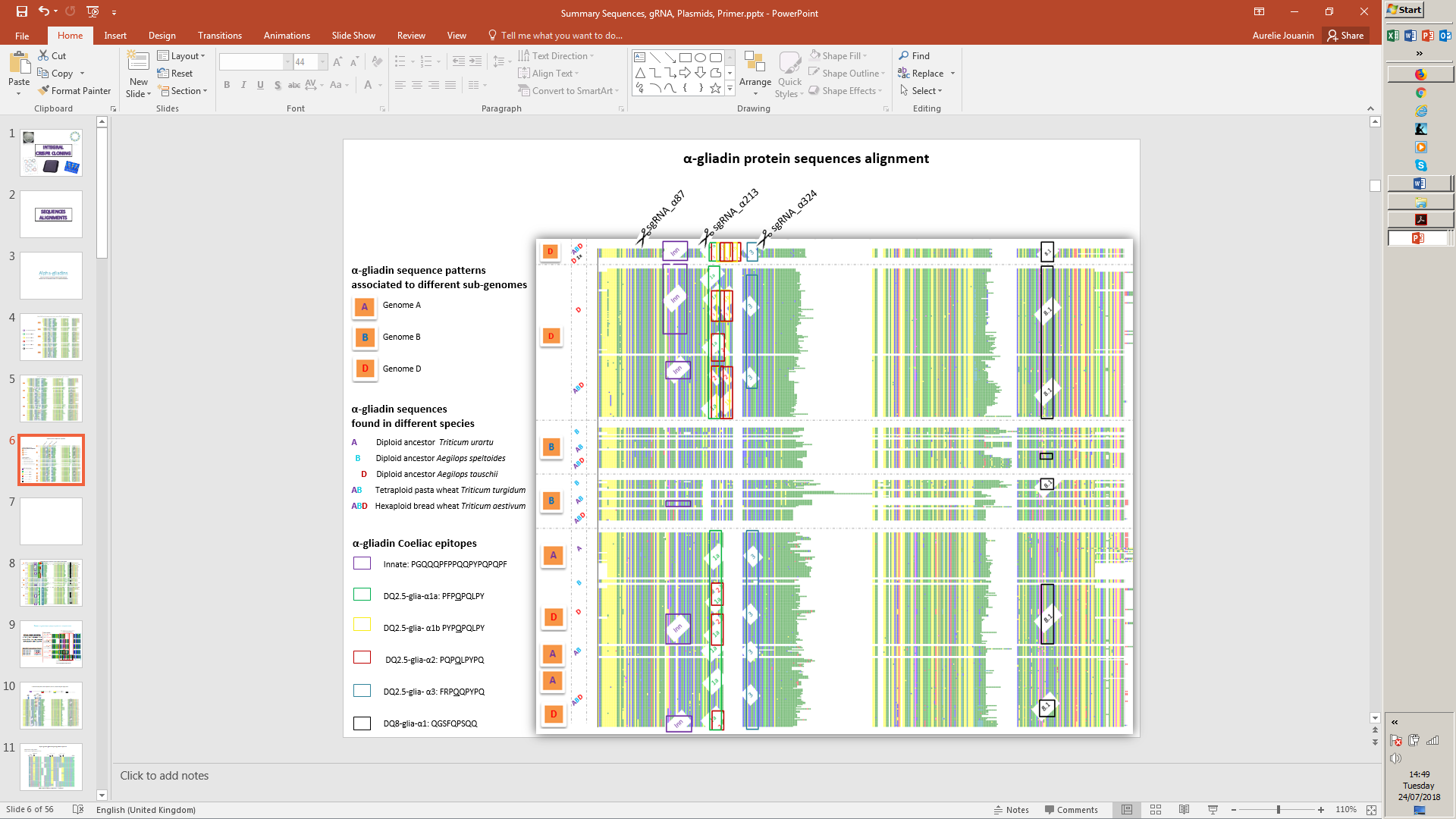


**genomes**

Supplement: Supplementary file 5 — α-gliadin protein sequences alignment. (DOCX 2533 kb) [file 12870_2019_1889_MOESM5_ESM.docx]

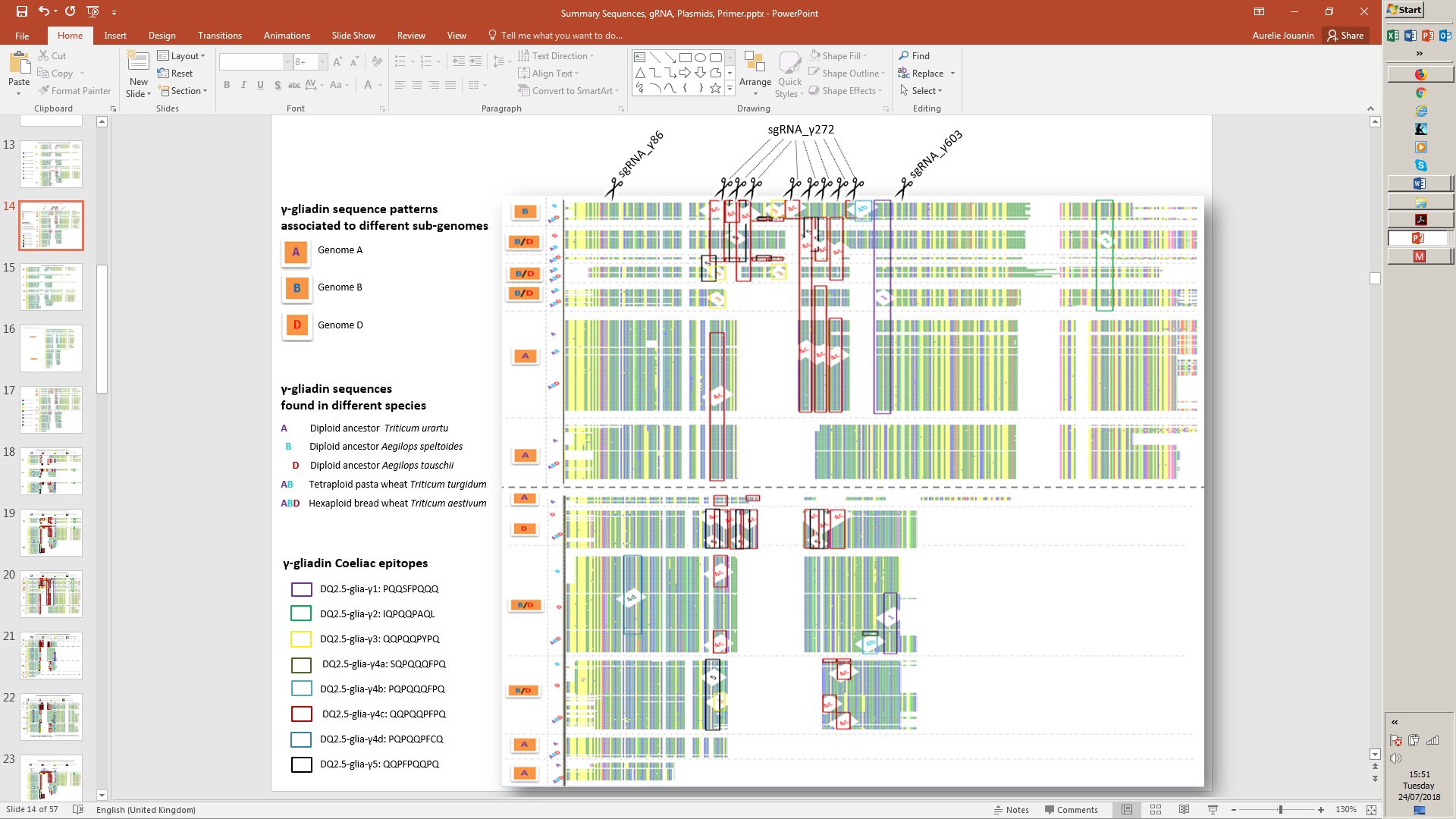


**genomes**

Supplement: Supplementary file 6 — γ-gliadin protein sequences alignment. (DOCX 2663 kb) [file 12870_2019_1889_MOESM6_ESM.docx]
